# Development of the EVIBEC Learning Outcomes Framework to support the delivery of evidence-based practice curricula in health care professional programmes: a codesign approach

**DOI:** 10.1186/s12909-023-04972-0

**Published:** 2024-01-03

**Authors:** Catherine Redmond, Robin Farrell, Catriona Cunningham, Andrea Dineen, Shane Foley, Deirdre O’Donnell, Sharleen O’Reilly, Diarmuid Stokes, Emma O’Neill

**Affiliations:** 1 UCD School of Nursing, Midwifery & Health Systems, Belfield, Dublin, Dublin 4 Ireland; 2 UCD School of Veterinary Medicine, Area: Veterinary Nursing, Belfield, Dublin, Dublin 4 Ireland; 3 UCD School of Public Health, Physiotherapy and Sports Science, Belfield, Dublin, Dublin 4 Ireland; 4 UCD School of Medicine, Belfield, Dublin, Dublin 4 Ireland; 5 UCD School of Agriculture and Food Science, Area: Dietetics and Human Nutrition, Belfield, Dublin, Dublin 4 Ireland; 6 UCD Library, Belfield, Dublin, Dublin 4 Ireland; 7 UCD School of Veterinary Medicine, Belfield, Dublin, Dublin 4 Ireland

**Keywords:** Evidence based practice, Healthcare professional education, Co-design, Inter-disciplinary education, Professional competency

## Abstract

**Background:**

All healthcare professional education programmes must adopt a systematic approach towards ensuring graduates achieve the competencies required to be an evidence-based practitioner. While a list of competencies for evidence-based practice exist, health care educators continue to struggle with effectively integrating the necessary competencies into existing curricula. The purpose of this project was to develop an open access cross-discipline, learning outcomes framework to support educators in integrating the teaching, learning and assessment required to ensure all graduates of health care professional programmes can achieve the necessary evidence-based practice competencies.

**Methods:**

An interdisciplinary team of health care professional educators and a librarian completed a review of the health professions literature on the teaching and assessment of evidence-based practice. The literature, coupled with the teams’ collective experiences in evidence-based education and research, were used to identify relevant teaching, learning and evidence-based competency frameworks to inform the project design. The guide and toolkit for experience-based co-design developed by the National Health Service Institute for Innovation and Improvement was adopted for this study ( Institute for Innovation and Improvement: Experience Based Design: Guide & Tools In. Leeds: NHS; 2009.). A four-step approach involving three online participatory co-design workshops and a national validation workshop was designed. Students (*n* = 33), faculty (*n* = 12), and clinical educators (*n* = 15) participated in formulating and mapping learning outcomes to evidence-based competencies.

**Results:**

Through a rigorous, systematic co-design process the Evidenced-based Education Collaborative (EVIBEC) Learning Outcomes Framework was developed. This framework consists of a series of student-centred learning outcomes, aligned to evidence-based practice competencies, classified according to the 5 As of EBP and mapped to the cognitive levels of Bloom’s taxonomy. Associated learning activities for each step of EBP are suggested.

**Conclusions:**

A consensus-based, student-centred learning outcomes framework aligned to a contemporary set of EBP core competencies has been developed. The freely accessible EVIBEC framework may support entry level health care professional EBP education, by informing EBP curriculum development and offering the potential for interdisciplinary approaches to and sharing of valuable teaching and learning resources. Co-design proved an effective method in creating and refining this framework.

## Background

Evidence based practice (EBP) is defined as the integration of best research evidence with clinical expertise and patient values [1] It is recognised as fundamental for safe, high-quality healthcare and therefore should be an essential component of all healthcare professional (HCP) education programmes [2–5]. Despite this recommendation, one of the long-recognised barriers to EBP education is lack of consistency in EBP curricular content [6–8]. Recent reports also highlight that effectively embedding EBP throughout HCP curricula remains a particular challenge [9–12]. While academics are supportive of teaching EBP, barriers such as competing job demands, lack of faculty skills, knowledge, and confidence, or supporting curricula frameworks impede progress [12–17]. These academic barriers impact HCP student EBP competency and contribute to deficits that continue to undermine healthcare quality, safety, and patient outcomes [15, 18]. 

In Europe, in 2005, an international working group of EBP health care educators and developers published the Sicily consensus statement on EBP [19], comprising a five-step, 5As model (Ask, Acquire, Appraise, Apply, Assess) for teaching and conducting EBP, and a description of core competency requirements. Since then, various EBP competency frameworks or statements have been developed independently by most HCP disciplines and their regulatory bodies [12, 20]. Despite this, the lack of uptake by healthcare educators suggests they may not meet the needs of all disciplines or educators [14, 21]. Additionally, where EBP has been integrated, inconsistencies in curricular content and the quality of teaching have been noted [8, 22]. Aiming to standardise and further enhance teaching, Albarqouni and colleagues [22] in 2018 developed an international, consensus-based, set of core EBP competencies for entry-level HCP programmes. This approach offers a universal shared language with clear benchmarks to inform EBP curricular development. 

Whilst defining a set of core competencies for EBP education was a necessary first step for the implementation of effective EBP education, such competency frameworks and statements provide a broad overview only. They do not identify in any detail the knowledge, skills, and attitudes (KSAs) required to be an effective evidence-based practitioner or articulate these as actionable learning outcomes. Since the Bologna agreement [23], outcomes-based curriculum design has increasingly been recognised as the appropriate approach for all third level institutions [23, 24]. Learning outcomes are defined as: “*statements of what a learner is expected to know, understand and/or be able to do at the end of a period of learning*” [25], and place the emphasis on student achievement of learning rather than content delivery [26]. 

The next logical step towards supporting EBP educators is to disaggregate the competencies of Alberqouni et al. [22] into a series of transparent and explicit achievable learning outcomes. These learning outcomes should encompass multiple cognitive learning levels allowing scope for progression and vertical integration of EBP learning from early to late programme stages. In addition, they should also be expressed from a student perspective in a way that students can clearly relate to, requiring a development process that is most effectively achieved using a co-design process [27–30]. Given that EBP competencies are required by multiple HCP disciplines, there is huge potential gain from an interdisciplinary approach to curriculum design, with the collaboration of an interprofessional team of HCPs offering additional benefits [31]. 

The overall aim of this study was to develop the first cross-discipline, vertically integrated, open -access, learning outcomes framework to support EBP curriculum development and delivery across entry-level HCP programmes. 

### Objectives

1. To articulate student-centred EBP learning outcomes
2. To create an EBP learning outcomes framework.
3. To identify student-friendly learning activities to attain EBP learning outcomes.

## Methods

An interdisciplinary (ID) group of academics (8 educators from six HCP programmes and one librarian), from a leading Irish University, involved in the design and delivery of Evidence Based Practice in their respective HCP programmes established the Evidenced-based Education collaborative (EVIBEC) project management team. Ethical approval for the project was obtained from University College Dublin, Human Research Ethics Committee (LS-E-20–166-Redmond). Access and support for the recruitment and involvement of registered students was obtained from the Heads of Schools/Disciplines in each of the participating healthcare programmes.

### Co-design approach

A co-design approach was deemed most suitable for the development of this learning outcomes framework. Co-design, within the context of health services research, is the process of bringing together service users, clinical and non-clinical staff, and at times, relevant support, and advocacy groups to work collaboratively to improve or refine elements of the care system, services, or processes [32]. This overall approach lends itself well to application in a healthcare education setting although reports of its use in this context are limited. What is clear from the few studies undertaken is that co-design in health professional education has great potential to provide a structured approach to collaborative working in creating and refining curriculum which is responsive to the needs and interests of students, educators, practitioners, and patients [28, 29, 33].

### Co-design team

The co-design team comprised the interdisciplinary EVIBEC project management team and thirty-three undergraduate and postgraduate students, registered to the participating HCP programmes in the University, (see Table 1).

**Table 1:** Co-design participant demographics

Group	Profession	Degree type	Year level within degree	Participants (n)
Students	Human Nutrition	Undergraduate	Year 4	4
	Dietetics	Masters	Year 2	4
	Physiotherapy	Undergraduate	Year 4	3
		Masters	Year 2	1
	Medicine	Undergraduate		2
		Graduate degree		2
	Nursing and midwifery	Undergraduate	Year 4	3
		Graduate degree		2
	Veterinary medicine	Postgraduate	-	2
		Undergraduate	Year 4	2
	Veterinary nursing	Postgraduate	-	2
		Undergraduate	Year 4	2
	Radiography	Undergraduate	Year 4	4
Academic members	Dietetics and human nutrition			3
	Nursing and midwifery			2
	Veterinary medicine			1
	Veterinary nursing			1
	Radiography			1
	Physiotherapy			2
	Library and information services			1
	Medicine			1

HCP students in the later stages of their programmes, who had undertaken mandatory EBP or research methods training within the last year, were sent an email outlining the study. Students responding to the initial call for expressions of interest were given an information leaflet and consent form for participation in the co-design team.

### Supporting meaningful involvement

Open, reciprocal, and democratic dialogue where all participants contribute equally is core to meaningful involvement in co-design [34]. When developing the co-design approach, the development team were mindful of the contextual, environmental, and social enablers to effective and meaningful participation [34, 35]. The team recognised the importance of fostering a positive team atmosphere which is receptive to the contribution of students. The strategies employed to ensure meaningful co-design included the identification of a faculty co-design team member as an ‘involvement champion.’ This champion was a named point of contact for the students if they had any queries or concerns that they wished to address. We also provided pre-workshop information sessions to ensure students felt confident when joining the wider co-design team. At these sessions we described the overall aim of the co-design process, discussed the roles of different co-design team members, reviewed the context and background for EBP education in healthcare professional programmes and introduced all the co-design team members on a first name only basis. Following best practice for co-design [34, 35], we ensured that there was a critical number of students from each of the 7 HCP disciplines recruited (min. 4 students from each discipline). This meant that students outnumbered faculty representation for each discipline and a student was never the sole student voice representing a discipline in any of the breakout groups. The aim of student recruitment and the co-design sessions was the amplification of the student voice relative to the faculty voice as well as balancing the representation across disciplines. Towards this aim, we made an explicit commitment to avoid use of jargon and to explain any technical language as and when it arose in discussions. The collective aim was for a relaxed and informal atmosphere with all team members identified by first name only.

Because COVID-19 public health guidance was in operation at the time of the co-design sessions, all workshops were conducted via Zoom™ (Zoom Inc, CA USA). Students were provided with an ‘icebreaker’ session in advance of the first workshop which ensured access, familiarity and confidence with virtual platforms being used. To accommodate the varying schedules of students across programmes all workshops were conducted in the evening. This facilitated attendance of students who were undertaking clinical practicums with resulting stable participation (bar one student who missed one workshop due to family commitments) throughout the workshops. All students received an honorarium (one-for-all voucher for €30) at the end of the project in recognition of their contribution to the co-design work.

### Research design

**Fig. 1:**
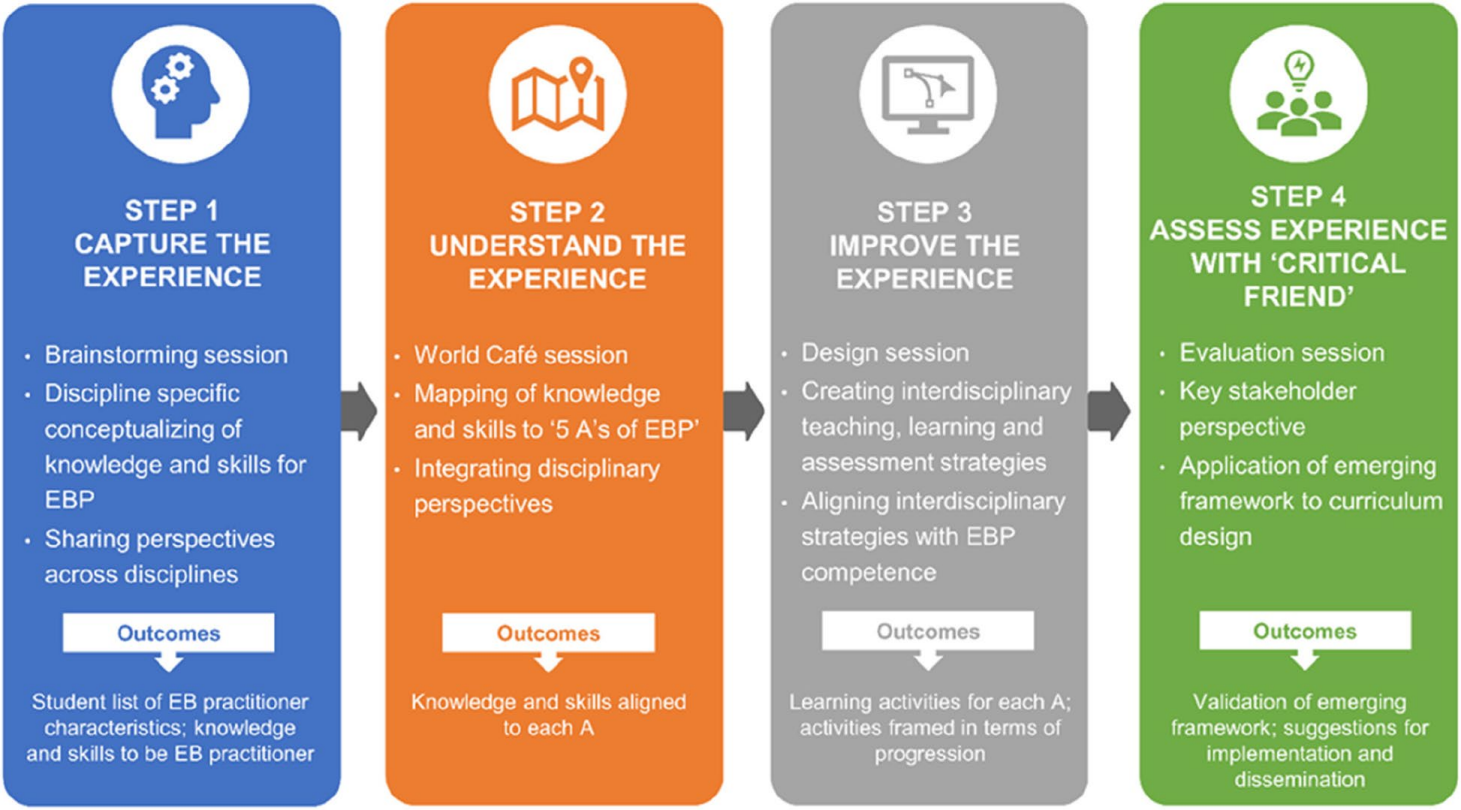
The four step co-design approach and target outcomes. Four iterative steps were taken, and a series of target outcomes were proposed for each step of the co-design approach

This study followed the guide and toolkit for experience-based co-design developed by the NHS Institute for Innovation and Improvement [36]. The strategies for meaningful co-design, described above, were embedded into a four-step iterative approach which started with an exploration of educator and student experience (step 1), to understanding these experiences (step 2), to improving the experience (step 3) and finally to assessing the experience (step 4). At the end of each step, data were collated and presented to students prior to the next step. These steps were taken during three online interactive and participatory co-design workshops and one key national stakeholder validation of the emerging framework event. Figure 1 describes each of the four experience-based co-design steps and illustrates the incremental nature of the work where the outcomes from each step provided the starting point for the next step. The online workshops were conducted using Zoom™ web conferencing platform and interactive collaboration was facilitated using Mural™ (Tactivos Inc. Buenos Aires, Argentina), a digital whiteboard and innovative platform for creative collaboration.

#### Step one: capture the experience

Capturing key stakeholder (here educators and students) experiences is identified by the NHS Toolkit as a critical first step in this co-design approach. This guide notes that the approach should start by helping people to tell the story of their experiences.

##### Step 1a) Educator experience of EBP

The EVIBEC project management team began with a series of brainstorming sessions exploring their experiences of facilitating EBP teaching, followed by a critical review of the literature to identify relevant teaching, learning and research frameworks to inform the project design. Albarqouni et al. [22] set of core EBP competencies for health professionals was deemed the most suitable for this project as these are contemporary and consensus-based and have recently been advocated by the Clinical Effectiveness Unit, Department of Health, Ireland, as relevant for all entry level health professional programmes [37]. Bruner’s spiral curriculum design was identified as appropriate to underpin the draft learning outcomes framework, where knowledge and skills are introduced and applied at increasing levels of complexity [38]. This type of spiral curriculum design supports student-centred outcomes-based education and provides flexibility in implementation. In addition, it was decided that Bloom’s taxonomy would provide the cognitive levels necessary to scaffold the progression of learning outcomes throughout all stages of a programme [39].

##### Step 1b) Student experience of EBP

The focus of this step was to assist students in telling the story of their own experiences of learning EBP. In the first co-design workshop, students were grouped by discipline in virtual break out rooms. Each room had a member of the project team with experience in facilitating group discussions. The Mural platform contained instructions on each task set, the 5 As and the core competencies. It also displayed a clock, so tasks were completed on time.

The tasks were designed to return students to educational experiences obtained in their previous EBP module (to be situated), facilitating them in processing how they thought it connected with their HCP practice. Task one required students, individually and then in their group, to reflect on what they considered to be the key characteristics of an evidence-based practitioner. This helped them to link back to their experience and facilitated them in discussing and exploring their individual and shared experiences and understandings with colleagues. Task two asked participants to outline the knowledge and skills they considered necessary to become this effective evidence-based practitioner. Finally, the group was asked to reflect on their experiences of EBP education and if, how and why they thought their educational experience had facilitated their acquisition of the knowledge, skills, and attributes that they had identified.

Following the small discipline group discussions, everyone returned to the main virtual room and shared their discipline group’s perspectives with the larger group.

At the end of this workshop all participants had:

- Developed discipline specific conceptualisation of competencies for EBP
- Experienced sharing of perspectives and commonalities across disciplines

#### Step two: Understand the experience

The overall focus of this step was to facilitate co-design participants in integrating their experiences of EBP learning with conceptual frameworks for the five steps of evidence-based practice.

Workshop two aligned with the principles of the world cafe method (https://theworldcafe.com/key-concepts-resources/design-principles/). This method has been used extensively in education to facilitate large group dialogue and has, more recently, been used in co-design health education projects [40]. It is characterised by targeted small group rotating discussions, with each conversation building on the last. Mural proved an ideal platform for virtual hosting this method. Participants in this virtual world café method were randomly assigned to 1 of 5 mixed-discipline groups and initially allocated to an outer ring of one of the 5A steps of EBP of: Ask, Acquire, Appraise, Apply or Assess. They were asked to reflect on, discuss, and using post-its, develop a list of knowledge and skills relevant to the 'A' assigned to that room. They then rotated to the next A and the middle ring to explore and add to the comments of former participants in that outer ring. This sequence was repeated one further time facilitating collaborative dialogue among many students on each task set. The facilitator in each room prompted contribution, listened for patterns and insights, and harvested an overview of the conversations to share with the whole group during the concluding session of the workshop. This overall process ensured the integration of interdisciplinary perspectives and experiences.

Following step 2, the project management team examined and discussed the data obtained. Duplications were removed, data was placed under the correct ‘A’ domain if misplaced, and the content was converted to measurable learning outcomes, framed using an action verb followed by an appropriate object for that verb. The project team also ensured that each learning outcome was appropriately assigned and scaffolded to each cognitive learning level of Bloom's Taxonomy: knowledge, comprehension, application, analysis, synthesis, and evaluation.

#### Step three: Improve the experience

The step focuses on the facilitation of co-design participants in the creation of ideas for learning activities that support the development of the knowledge and skills identified in step 2.

Following principles from the participatory research method of deliberative dialogue, where the best course of action is determined through discussion [41, 42], in workshop three students were placed in interdisciplinary groups within breakout rooms. In these groups they discussed learning activities that they had previously found useful for learning EBP and proposed additional novel exercises that they thought might aid learning within each of the EBP A domains. They then framed these activities in terms of their position and progression within their programmes (early, middle or late-stages). At the end of the process, students were asked to reach a consensus, identifying one key Interdisciplinary learning activity for each ‘A’ and prepare a ‘sales pitch’ to present to the whole group explaining their choice. Each facilitator`s role was to absorb information, promote reflection, and provide feedback, encouraging prioritising with the intention of identifying and reaching consensus on the intervention components.

#### Step four: Assess the experience with a ‘critical friend’

In this final step of the co-design process the emergent co-designed framework was presented to an audience of stakeholders and disciplinary experts to allow open discussion and feedback. This workshop was delivered at the Irish Network of Healthcare Educators (INHED) online national conference in Dublin in 2021. Information re the workshop was made available on the conference website, outlining who it would be of interest to, that workshop data would form part of an on-going research project, that ethical permission for the study was obtained from the hosting institution and that informed consent would be gained from all conference participants on registration to the workshop. Participants (*n* = 15) were also asked to complete a short demographic survey.

During this interactive workshop, participants undertook some of the same exercises that had been performed by students in their workshops. The aim of this was to provide context for the participants and to explore their perceptions of the knowledge, skills and learning strategies necessary to attain the 5 A EBP competencies. In break out rooms participants then explored the developing EVIBEC learning outcomes framework and its related learning activities and were asked to validate or contest its content. All discussions were fed back to the whole group at the end of the session to derive a consensus.

Finally, the participants in this 4th workshop brainstormed immediate and long-term suggestions for implementation and dissemination of the EVIBEC framework under the following headings: clinical practice; module/ course level; programme level—positioning, embedding, acceptability, feasibility, training, evaluation; Institution level—policy and procedures; National level—regulatory bodies, dissemination mechanisms and international level**.**

## Results

### Participants

The demographic profile of the student and educator participants is presented in Table 1. A minimum of four students participated from each of the disciplines involved, with some registered to late stages of an undergraduate degree and some registered at postgraduate level. Each discipline had an educator team member participating.

### The EVIBEC learning outcomes framework

The EVIBEC Learning Outcomes Framework was produced following the iterative four step co-design approach and the target outcomes were achieved for each step (Fig. 1). The framework is the result of a collation of the work from educators, students, and participants from the INHED conference. Student and conference participant data were collected in the format of virtual sticky notes from their mural boards and summary documents created by each of the room facilitators. Due to time constraints and lack of student familiarity with learning outcome development, student data was not written in the form of learning outcomes. Instead, sticky notes consisted of short terms or single words, indicating the knowledge and skills students perceived essential for an effective evidence-based practitioner. For example, in step 2, short statements such as *“know how to perform a search”* or *“use Boolean operators' ' or “which CASP tool?”* were received.

Step 3 outcomes comprised a list of innovative non-traditional learning activities designed to meet the learning outcomes. These have been incorporated into the EVIBEC learning outcomes framework as separate Padlet pages [43]. Overall, students actively favoured contextual and situated learning activities as might be expected of adult learners [44–46]. Gamification of learning activities were proposed such as *“Who`s who: what source are you”* and *“navigating virtual cases with graduated information release.”* In the discussions students voiced their opinion that inclusion of a gaming component improves motivation. They also added that incorporating conceptual levels of difficulty or “cognitive scaffolds” into activities, similar to performance levels found in gaming, promote learning, and keep learners engaged. Students favoured peer-learning and interactive teaching strategies such as “*final years presenting to 1st years*” and “*group case discussions using online forums*”. They were also explicit about beginning to learn EBP early in their programmes with the incorporation of EBP throughout different modules and all stages. However, notably only one group identified teaching strategies that incorporated EBP in clinical placements: “*be provided with a tricky clinical dilemma—what's the evidence to guide practice—can be in clinical or classroom setting.”*

The final step occurred during the INHED conference. In this workshop participants were clinical HCP educators. They also suggested EBP learning activities, however these tended to focus on learning in clinical practice. They advocated for active learning strategies, problem-based learning, and work-based learning approaches. Suggested activities included: the practical use of audit tools; sharing examples; having a repository of “real” examples and having patients involved. They also agreed that the purposeful teaching of EBP should be positioned throughout healthcare curricula, within practice placements in addition to multiple academic modules. To aid this it was suggested that stronger links be made between academia and clinicians, with a two-way sharing of resources. The project team later reviewed suggested activities from workshops 3 and 4 and assigned them to the appropriate ‘A’ domains of the evolving learning outcomes framework.

Steps 1–4 resulted in the generation of the preliminary EVIBEC Learning Outcomes Framework. The framework can be found on https://www.ucd.ie/chas/research/evibec/. It is structured with the 5 As presented as columns from left to right across the screen and the colour coded cognitive levels of Bloom’s taxonomy as rows. Learning outcomes have been added to each column. Additional screens can be accessed from the main page, with a link at the top of each column providing access to a page for each ‘A’ and one at the bottom of each column providing a link to suggested activities for each A, derived from the workshop participants.

It must be stressed that this framework is evolving as EBP experts engage and contribute to it. The framework is hosted on Padlet (San Francisco, California, U.S.), a cloud-based collaborative platform designed to act like a virtual notice board, allowing real-time interaction and sharing of materials. This approach was selected as it allows what would have been a very large and unwieldy table to become easy to navigate and find the relevant sections. It also offers huge potential for open access sharing of materials and ideas, and it is hoped that it would form the basis to establish a community of practice approach to support validation and refinement of the framework. An iterative approach involving different stakeholders and experts from other disciplines and countries is essential and welcomed and will drive this refinement.

## Discussion

Through a rigorous, systematic co-design process, involving an interdisciplinary group of HCP educators and relevant student groups, the EVIBEC framework is presented for the first time. This framework consists of a series of learning outcomes, aligned to EBP competencies, classified according to the 5 As of EBP and mapped to the cognitive levels of Bloom’s taxonomy. The framework is designed to support the integration of teaching and learning related to evidence-based practice throughout all entry level HCP programmes. There is clear value in designing an interdisciplinary syllabus for EBP. Firstly, it has core relevance across health professional programmes with very similar approaches relevant to each and hence significant potential gains from the sharing of resources [8, 19]. EBP competencies transect the healthcare disciplines regardless of the scope of practice and additionally feature heavily in the learning outcomes and pedagogical approaches within individual disciplines, with a common overall goal of effective EBP. Secondly, and of significant importance, as many professionals practise as a team, a trans-curricular approach will equip students with shared EBP language, knowledge, and skills. This will facilitate their future engagement in multi-disciplinary teams, debates, and practice development, all progressing an effective, constructive EBP agenda and future alignment of the professions.

The EVIBEC learning outcomes framework was developed with recognition of the requirement for progression of learning from early to late programme stages, a requirement articulated strongly by both students and educators. A programmatic approach with deliberate progression of learning using scaffolded delivery approaches should enhance student EBP skill development, allowing students to build on learning gained during previous and parallel modules [27]. The explicit detailing of the knowledge, skills and attitudes required for competency in each of the 5 As should enable HCP educators to recognise and appreciate critical elements of EBP and should in turn afford greater consistency and transparency in curricular content. This framework is one that will support both entry level HCP education and continuous professional development for HCPs in clinical practice, especially important where HCPs undertook their professional education at a time where less emphasis may have been placed on EBP. Now hosted on the freely accessible website this framework will support educators in adopting a more systematic and explicit approach to the implementation of EBP learning in HCP programmes. It is also designed as a mechanism to promote the sharing of resources and the development of a community of practice aligned to the common aim of enhanced interprofessional EBP teaching and learning.

The underpinning beliefs of the interdisciplinary project team led to the choice of a co-design approach to this framework. In agreement with Moriña [47] and O`Connor [48] the team were of the belief that students have a valuable contribution to make and represent a valuable source of knowledge and insight for the effective development of teaching and learning resources. Participatory co-design effectively fosters negotiation, participation, and inclusion of the voice of the students [49]. As end users of teaching pedagogies, it is recommended that students should be co-collaborators in the development and evaluation of teaching and learning approaches to ensure an effective user-centred design [50]. In this study students appreciated being invited to contribute to enhancing curriculum and felt valued and empowered by this process. As one student voiced*: “workshops were very useful and the dialogue beneficial to give a voice to and feel heard.”* The student perspective provided unique insights—students were comfortable outlining the knowledge and skills required for the early EBP steps of Ask, Acquire and Appraise. However, in line with findings from other studies [22, 51], they struggled to express the components of Apply and Assess. They also required facilitator prompting to consider the connection between shared decision making, patient preferences and EBP. This difficulty of expressing progression has been described as the novice-expert divide where experts in a domain have attained and organised a large amount of information and may not appreciate the step-by-step learning needs of novice or advanced beginner students [44]. Co-creating a scaffolded learning outcomes framework with students moving from lower-level learning outcomes before progression to higher level learning was very instructive for both parties. It facilitated the incorporation of important learning outcomes that may have otherwise been overlooked by the project team. It also provided clear insight to educators about areas students find more challenging and in turn how best they can scaffold the curriculum to meet the student needs.

An important element of this study`s co-design process concerns the general features of student suggested learning activities. The students emphasised their preference for a wide range of contextual activities and emphasised that these had to involve active contextual interactions as this met a broader range of student learning preferences. They also suggested activities that would trigger their curiosity and enhance their motivation to learn. This is in line with other studies showing that students value access to different kinds of resources to support, motivate and enhance their learning with an adult learner preference for 'real life' learning [45, 46]. The diversity of the student group and their disciplinary differences enriched these discussions exposing participants, both educator and student alike, to a rich variety of learning experiences and activities. As voiced by one student “*the interdisciplinary approach allowed us to get out of our own profession’s ‘bubble’ and think in new /alternative ways".* The awareness of ideas and approaches from different disciplines also widens the variety over a single disciplinary approach.

The voice of clinical educators is equally valuable as their experiential knowledge is important in helping academics acknowledge the “multiple realities and meanings” of EBP in action [29, 52]. HCP educators in workshop four provided important insight into how learning outcomes and suggested aligned learning activities might support the learning and application of EBP knowledge in different learning spaces. There was a stark contrast between the focus of HCPs on learning in the clinical context, while students focused on learning in the HEI. Lewin et al. [53] argue collaboration and co-design opens new ways to connect and combine different learning sites in higher education in which both universities and clinical institutions are equal partners in influencing learning and competence development in students. This certainly appeared evident in this study. Clinical educators welcomed being collaborators in the development of the EVIBEC framework, ensuring the inclusion of learning outcomes which cross clinical-HEI boundaries The setting up of mechanisms to facilitate sharing of online resources was seen as a pragmatic example of this partnership as they identified their unique ability to share the practical use of audit tools, to share a repository of “real examples” and databases of evaluations of patient and practice outcomes. They also suggested joint professional development sessions for academics and clinicians to facilitate theory practice transfer and appointments of adjunct professor positions. This type of collaboration fosters seamless learning processes thus enabling students to navigate between different spaces and different roles promoting and developing transferability between learning contexts in HCP education and decreasing the theory–practice gap [54, 55]. It is important that HEIs focus on EBP learning as part of clinical/professional work placement education and future developments and implementation should occur in close collaboration with students, the university, and the clinical setting thus maximising the overall integration of EBP education.

### Limitations

The framework was developed within a single university in Ireland, a situation counterbalanced by external stakeholder feedback from the INHED conference and several validation exercises by way of conference presentations, an Irish National Forum seminar, and the seeking of expert opinion from *EBP Ireland*. Further validation and refinement will be sought from other experts and international colleagues. Participating students in this study were self-selected and might not necessarily represent the full diversity of the university’s student population. The university has a wide range of representative HCP educational programmes mapping to the One Health agenda, but some HCP disciplines were not represented (e.g., Dentistry, Pharmacy, Occupational therapy, etc.) which may limit the study’s generalisability. However, the aim of hosting the EVIBEC framework on the Padlet platform is that it will establish a community of practice and ongoing framework iterations. Further external validation is warranted, and the patient voice could also be sought to further progress this collaborative approach.

## Conclusions: brief summary and potential implications

Combining student and clinical educator experiential knowledge with scientific knowledge gained from a literature search and the EVIBEC team’s expert EBP knowledge facilitated the development of a shared understanding of EBP and the development of this consensus framework. The freely accessible EVIBEC framework will support entry level HCP EBP education, offering the potential for interdisciplinary approach development and sharing of valuable resources. The overall goal is the support of evidence-based healthcare with a view to enhancing healthcare efficiency and effectiveness. The co-design process involving HCP educators and students proved highly successful in development of the EVIBEC learning outcome framework.

## Data Availability

The datasets generated and/or analysed during the current study are available from the corresponding author on reasonable request.
